# Staphylococcal Infections and Neonatal Skin: Data from Literature and Suggestions for the Clinical Management from Four Challenging Patients

**DOI:** 10.3390/antibiotics12040632

**Published:** 2023-03-23

**Authors:** Domenico Umberto De Rose, Flaminia Pugnaloni, Ludovica Martini, Iliana Bersani, Maria Paola Ronchetti, Andrea Diociaiuti, May El Hachem, Andrea Dotta, Cinzia Auriti

**Affiliations:** 1 Neonatal Intensive Care Unit, “Bambino Gesù” Children’s Hospital IRCCS, 00165 Rome, Italy; 2 Dermatology Unit and Genodermatosis Unit, “Bambino Gesù” Children’s Hospital IRCCS, 00165 Rome, Italy

**Keywords:** bullous impetigo, Staphylococcal scalded skin syndrome, epidermolysis bullosa, burns, *Staphylococcus aureus*

## Abstract

Staphylococcal infections in neonates might be associated with skin blistering since early antibiotic therapy has been shown to limit infection spread and positively influence outcomes; therefore, neonatologists should be aware of these conditions. This review examines the recent literature on the management of Staphylococcal infections that involve neonatal skin, discussing the most appropriate clinical approach to four cases of neonatal blistering diseases with Staphylococcal infections: a case of Staphylococcal bullous impetigo, a case of Staphylococcal scalded skin syndrome (SSSS), a case of epidermolysis bullosa with overlapping Staphylococcal infection, and a case of burns with overlapping Staphylococcal infection. In treating Staphylococcal infections involving skin in neonates, the presence or absence of systemic symptoms should be considered. In the lack of evidence-based guidelines in this age category, treatment should be individualized according to several factors including the extension of the disease or further skin comorbidities (such as skin fragility), with a multidisciplinary approach.

## 1. Introduction

Staphylococcal infections can be the leading cause of skin blistering in neonatal age [1]. It is crucial to be aware of newborn infections that might produce blistering since early antibiotic therapy has been shown to limit infection spread and positively influence outcomes [2].

*Staphylococcus aureus* (*S. aureus*) is a major human pathogen that causes many illnesses, from minor skin infections to life-threatening bacteremia. It contributes to community-associated and healthcare-associated infections, with a significant clinical burden for newborns globally [3]. The selection of Methicillin-resistant *Staphylococcus aureus* (MRSA) strains is increasingly becoming problematic in neonatal intensive care units (NICUs), with 6.7% of infants that become MRSA colonized and 2.0% of infants that had an MRSA infection according to data from the United States [4]. In particular, the increased survival rate of extremely preterm newborns may lead to an increasing group of infants at risk for MRSA colonization and subsequent infections [5]. MRSA surveillance may protect non-MRSA colonized neonates from becoming colonized. This is of considerable importance because the acquisition of colonization during hospitalization was associated with a 10-fold increase in the risk of developing MRSA bloodstream infection [6].

Coagulase-negative staphylococci (CoNS) (such as *Staphylococcus epidermidis, Staphylococcus hominis*, and *Staphylococcus haemolyticus*) are the bacteria most frequently recovered from blood cultures in neonates. However, it is not always clear whether CoNS represents a true infection or just a contaminant normally present on the skin [7]. The clinical importance of these bacteremia occurrences is often questioned, although clusters of CoNS bacteremia with considerable morbidity and difficulty in eradication can be observed in neonates, despite in vitro antibiotic susceptibility [8].

The current gaps in this topic in the published literature include the lack of evidence-based recommendations about the role of antibiotic treatment in neonates with Staphylococcal infections that involve neonatal skin, with the potential risk of progression to more severe forms. Therefore, this review examined the recent literature on the management of Staphylococcal infections that involve neonatal skin, discussing the most appropriate clinical approach to four cases of neonatal blistering diseases with Staphylococcal infections: a case of Staphylococcal bullous impetigo, a case of Staphylococcal scalded skin syndrome (SSSS), a case of epidermolysis bullosa with overlapping Staphylococcal infection, and a case of burns with overlapping Staphylococcal infection.

## 2. Results

A total of 23 records were identified through the literature search (via PubMed): of them, only 18 papers were written in English. Among them, 16 were excluded based on the titles and the abstracts. Thus, two papers were initially considered.

The first was a retrospective study by Markham et al. of infants hospitalized with skin and soft-tissue infections (SSTI), in which a wide variation in empirical antibiotic selection has been noted: clindamycin was the most commonly used staphylococcal antibiotic, whereas 21.4% of patients received vancomycin. However, they did not discuss the management of different conditions because the aim of the study was to describe empirical antibiotic selection in infants with SSTI across 36 children’s hospitals and investigate the association of different empirical antibiotic regimens with the length of hospital stay, readmission rates, and costs [9].

In the second study, Britton et al. retrospectively found that among pediatric community-associated *Staphylococcus aureus* infections, one-fifth were due to MRSA. However, they did not discuss the management of different conditions because this was not the aim of their study [10].

Therefore, since it was not possible to include these two articles, we considered for each paragraph additional studies that were identified based on our knowledge of the field or searching via PubMed. Forty-eight articles were identified by the search “Staphylococcal bullous impetigo” AND “neonate”; two-hundred and seven articles were identified by the search “Staphylococcal scalded skin syndrome” AND “neonate”; twenty-three articles were identified by the search “Staphylococcal infection” AND “epidermolysis bullosa” AND “neonate”; forty-one articles were identified by the search “Staphylococcal infection” AND “burns” AND “neonate”.

## 3. Materials and Methods

We described four cases of neonates with Staphylococcal infections, recently admitted to the neonatal intensive care unit of “Bambino Gesù” Children’s Hospital. The first three cases were from January 2022 to January 2023, and the last case was hospitalized in 2015.

We searched PubMed for cohort, cross-sectional and case-control studies, reviews, expert consensus, case series, or case reports published as articles or letters to the editor describing neonates with Staphylococcal skin infections. An extensive literature search has been performed up to 15 January 2023. The following keywords were searched as MeSH entry terms: “Staphylococcal infection” AND “infant, newborn” AND “skin”. We excluded all retrieved articles written in non-English languages. Additional studies were identified by authors based on their knowledge of the field, if not already included in the literature search. Parents signed a written informed consent form regarding publishing their data and photographs.

## 4. Staphylococcal Bullous Impetigo

### 4.1. Patient

**Figure 1:**
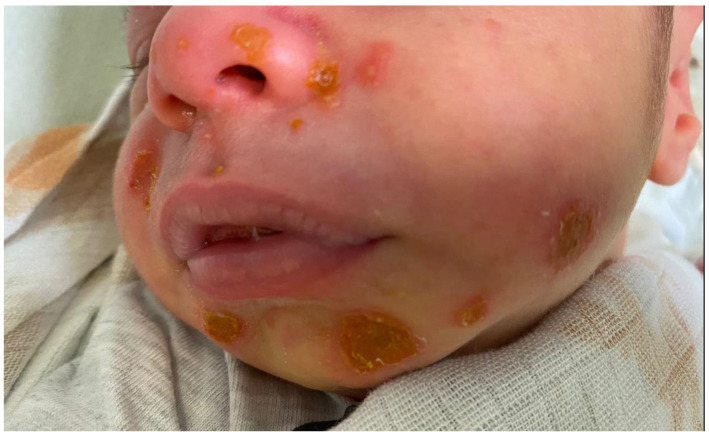
Erosions in a Staphylococcal bullous impetigo in a neonate.

A 10-day-old male newborn was admitted to our unit with irritability, poor feeding, and maculopapular lesions, but without fever. The skin lesions were 10–15 in number, characterized by honey-colored crusts, and distributed over the face, sparing the trunk, extremities, palms, and soles. The mother reported that in the previous two days, the maculopapular lesions transited into small vesicles and blisters that rapidly ruptured, leaving erosions covered by honey-colored crusts (Figure 1).

No relevant prenatal history was revealed. The baby was born by vaginal delivery after 40 weeks of an uneventful pregnancy from a healthy mother. Birth weight, length, and head circumference were 3560 g (60th centile), 52 cm (80th centile), and 35 cm (59th centile), respectively. Vagino–rectal swabs before delivery were negative. No history of sexually transmitted infections or genital lesions was revealed.

Laboratory examinations at admission showed a whole blood count of 11,080 cells/mm^3^ (normal values: 6000–21,000 cells/mm^3^) with 32% neutrophils and 49% lymphocytes, hemoglobin of 16.5 g/dL (normal value: >12 g/dL), platelet count of 338,000/mm^3^ (normal values: >150,000/mm^3^), C-reactive protein (CRP) of 0.03 mg/dL (normal value: <0.5 mg/dL), and procalcitonin (PCT) was 0.07 ng/mL (normal value: <0.5 ng/mL). Electrolytes, liver, and renal function tests were normal. Nasopharyngeal, urine, and blood cultures were negative. A lumbar punction was not performed. Fungal blood cultures and skin swabs for pathogenic fungi were negative.

The swab of skin lesions was positive for *Staphylococcus aureus*. The organism was susceptible to oxacillin, clindamycin, vancomycin, and teicoplanin. Early treatment was started with intravenous ampicillin for 5 days with rapid improvement of the clinical conditions, feeding ability, and a significant decrease in skin lesions. Facial lesions were also topically treated with a 2% eosin solution for five days (Table 1). The baby was discharged home in good clinical condition after 6 days of hospitalization, and there was no recurrence of skin lesions within 20 days of subsequent clinical follow-up.

### 4.2. Management

Impetigo, a bacterial infection, involves the epidermis and the superficial dermis, with yellowish crusts on the face, arms, or legs that can cause discomfort or itching [11]. Non-bullous impetigo is the most common presentation due to *Staphylococcus aureus* or Streptococcus pyogenes [11]. The bullous form is caused by *Staphylococcus aureus* and is distinguished by huge bullae that can rupture and exude yellow secretions. *S. aureus* has emerged as the most common etiological pathogen, with an increasing incidence of community-associated methicillin-resistant (CA-MRSA) strains [12]. The differential diagnosis of blistering skin diseases in neonates includes contact dermatitis, herpes simplex infections, congenital Syphilis, neonatal pemphigus, candidiasis, and varicella zoster virus infections. Toxic epidermal necrolysis is a severe blistering eruption that involves the skin and mucous membranes; it is most often secondary to medications, and thus is rare in a neonate who received no drugs during the first days of life.

Rare complications due to the local or systemic spread of the infection include cellulitis, osteomyelitis, septic arthritis, glomerulonephritis, pneumonia, and sepsis [13,14]. The diagnosis is usually clinical, whereas causative pathogens can be identified only by culture or molecular identification of samples obtained by skin lesions. Nikolsky’s sign is negative (a skin finding in which the top layers of the skin slip away from the lower layers when rubbed). The growth of bacteria from culture is useful to assess antimicrobial susceptibility and guide antibiotic treatment, especially in the case of multidrug resistant strains [14].

There is no generally agreed standard therapy, and guidelines for treatment differ widely. Treatment options include many different topical and systemic antibiotics; it usually resolves within two to three weeks without scarring [15]. Topical treatment is always indicated and may be sufficient for limited impetigo extension (<2% of total body surface area) [14]. Among topical antibiotics licensed for treating bullous impetigo, fusidic acid and mupirocin are mostly used. Ozenoxacin is a topical antibiotic recently approved in treatment of non-bullous impetigo in adults and children aged ≥6 months, but it should be not used against the bullous form [16].

Systemic treatment is recommended in cases of extensive or multiple lesions and children < 1 year of age [14], with spread skin lesions and/or with parents not able to manage the treatment at home. Thus, especially in preterm neonates and term neonates with systemic symptoms, hospitalization and intravenous therapy represent the safest approaches to avoid complications. Indeed, our case of neonatal Staphylococcal bullous impetigo shows the rapid improvement of general conditions after the administration of parenteral antibiotics, avoiding the progression to SSSS. In Table 2, we summarized our personal opinions and recommendations about the management of these forms. Furthermore, impetigo in infants younger than 2 months can be associated with colonization by a maternal strain. Therefore, good hygiene and prevention measures should be recommended, such as the protection of bullous and excoriated lesions, in order to avoid the risk of self-infection and their spread to other areas of the body and mother-child pair decolonization [17].

## 5. Staphylococcal Scalded Skin Syndrome

### 5.1. Patient

**Figure 2:**
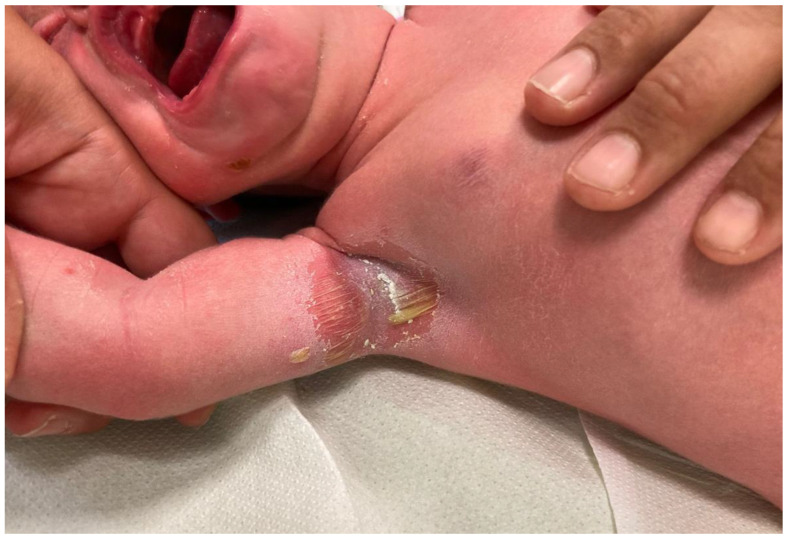
Case of SSSS in a neonate, with diffuse erythema and skin exfoliation at the level of the cubital fold and fluid-filled blisters that are thin-walled and easily ruptured.

A 7-day-old female newborn was admitted to our neonatal unit with skin lesions characterized by honey-colored crusts on the chin and exfoliative lesions in the axillary region bilaterally about 2 cm by 2.5 cm in size (Figure 2), with diffuse erythema, and discomfort but without fever. The oral mucosa was not involved. The mother reported that the lesions had appeared since the first days of life and the axillary lesions would have progressively increased in size. No relevant prenatal history was revealed. The baby was born by cesarean section after 41 weeks because of failure to start labor after an uneventful pregnancy. Birth weight, length, and head circumference were 3040 g (18th centile), 50 cm (44th centile), and 36 cm (93rd centile), respectively. The Apgar score was 9 and 10 at the 1st and 5th minute, respectively. Vagino–rectal swabs before delivery were negative. No history of sexually transmitted infection or genital lesions was revealed.

Laboratory examinations at admission showed a whole blood count of 11,680 cells/mm^3^ (normal values: 6000–21,000 cells/mm^3^) with 22.8% neutrophils and 55.6% lymphocytes, hemoglobin of 17.4 g/dL (normal value: >12 g/dL), platelet count of 359,000/mm^3^ (normal values: >150,000/mm^3^), CRP of 0.17 mg/dL (normal value: <0.5 mg/dL) and PCT of 0.1 ng/mL (normal value: <0.5 ng/mL).

Electrolytes, liver, and renal function tests were normal. Urine and blood cultures were negative. A lumbar puncture was not performed. The swab of the lesion was positive for *Staphylococcus aureus*. The organism was susceptible to oxacillin and methicillin. Skin swabs for pathogenic fungi and herpes simplex virus types 1–2 were negative.

Early treatment was started with intravenous ampicillin. About 24 h after admission, the child showed a new vesicle lesion (0.5 cm by 0.5 cm in size) with serous content on the left shoulder, which quickly broke and resulted in an exfoliative lesion. Subsequently, no new lesions appeared. CRP and PCT remained negative during hospitalization. Antibiotic therapy was administered for seven days, and lesions were also topically treated with a 2% eosin solution (Table 3). The baby was discharged in good clinical conditions, and there was no recurrence of skin lesions within 20 days of subsequent clinical follow-up.

### 5.2. Management

Most cases of SSSS are caused by methicillin-susceptible *S. aureus* (MSSA), but vancomycin, linezolid, or other drugs effective against MRSA should be considered in areas with a high prevalence of methicillin-resistant *S. aureus* (MRSA). Staphylococcal scalded skin syndrome (SSSS), also known as Ritter disease, is a potentially life-threatening disorder, more common in newborns than in adults, caused by certain strains of *Staphylococcus aureus* releasing serine protease exfoliative toxins. All strains of *S. aureus* produce toxins, but only five percent of them (most often from the phage group 2, types 3A, 3B, 3C, 55 and 71) release the exfoliative toxins A and B (ETA and ETB, respectively) that cause SSSS [18,19]. ETA and ETB undergo hematogenous dissemination to the skin at distant sites; they cleave desmosomal cadherins, in particular desmoglein 1, in the superficial epidermis, disrupting keratinocytes adhesion, and causing blistering and skin denuding [19]. Desmoglein 1 is present in the superficial epidermis but not in the mucosal membrane. This explains why the mucosal membranes are not affected in SSSS [20]. The primary sources of infection in neonates are the umbilical area (omphalitis), the diaper area, and periorificial areas such as perianal, perioral, periocular, and nasal regions (e.g., pustules, impetigo, and cellulitis) [19,21].

Indeed, within one week of birth, approximately 30% of neonates are colonized by *S. aureus* strains. However, some studies have shown carriage rates of 60% to 90% in newborn infants discharged from hospitals, especially when there are staphylococcal outbreaks, and discourage the use of antiseptics for the umbilical cord care. A clinical staphylococcal infection is not always present, and the neonate can have a normal appearance. The incubation period from skin infection to the manifestation of the syndrome can range from 1 to 10 days [22].

The diagnosis of SSSS is based on the typical clinical features with a diffuse marked erythematous rash with significant tenderness, discomfort, a positive Nikolsky sign (the separation of the upper epidermis from deeper layers with the application of light pressure), and the absence of mucosal involvement [19]. In contrast to bullous impetigo, there are more often systemic signs and symptoms in SSSS forms; however, in neonates, the discomfort due to lesions and food aversion (also observed in milder forms of bullous impetigo) can make it difficult to provide the differential diagnosis between these two entities.

The diagnosis is clinical (see clinical criteria—Table 4) [23]. It can be confirmed by culturing *S. aureus* from any suspected primary focus of infection, such as the nasopharynx, conjunctiva, umbilicus, and diaper area. Culturing exfoliative lesions is not helpful since they are caused by staphylococcal exotoxins, and *S. aureus* is not usually present in the skin lesions. Indeed, the particularity of this case was the finding of MSSA strains in skin lesions. Positive blood cultures are uncommon (<15%) [23]. Patients with SSSS typically require hospital admission for intravenous antibiotic therapy and supportive care.

## 6. Staphylococcal Infections in the Neonate with Epidermolysis Bullosa

### 6.1. Patient

**Figure 3:**
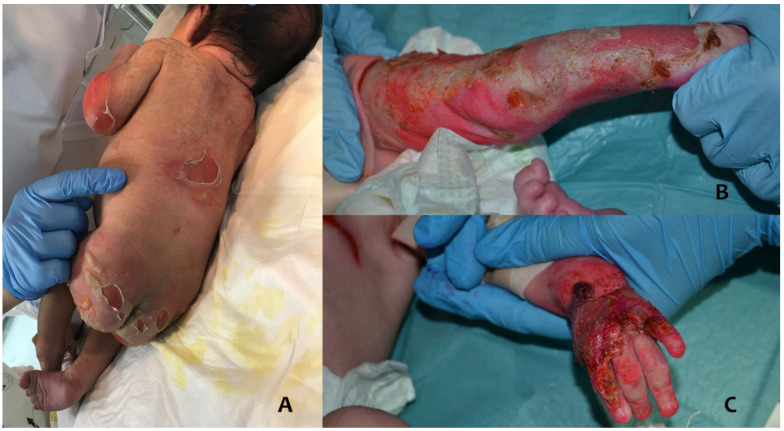
A case of a neonate with severe Junctional Epidermolysis bullosa (**A**), with Staphylococcal skin infection of the left leg (**B**) and the left hand (**C**).

A 6-day-old female newborn was admitted to our neonatal intensive care unit with skin lesions characterized by de-epithelialized areas on the buttocks (Figure 3A); extensor surfaces of the elbows and back with bullous serous lesions, surrounded by erythema; blisters with serous content on the auricles bilaterally; a crusted lesion of about 1.5 cm in diameter on the chin; erosion of the back of the 1st and 2nd finger of the right hand, and of the first four fingers of the left hand; and nail dystrophy of the 2nd finger of the right hand. At birth, the lesions on the hands and face were already present. Given the rapid deterioration of the skin condition, the newborn was transferred to our hospital on suspicion of epidermolysis bullosa.

The baby was born at term by vaginal delivery after an uneventful pregnancy at 39 weeks of gestational age. Birth weight was 3.130 g (31st centile). The Apgar was 9 and 10 at the 1st minute and at the 5th minute, respectively. The baby was the second child of consanguineous (first cousins) healthy parents from Bangladesh, with no reported family history of remarkable diseases. Vagino–rectal swabs before delivery were negative.

Laboratory examinations at admission showed a whole blood count of 8900 cells/mm^3^ (normal values: 6000–21,000 cells/mm^3^) with 52.6% neutrophils and 19.4% lymphocyte), hemoglobin of 17.3 g/dL (normal value: >12 g/dL), platelet count of 310,000/mm^3^ (normal values: >150,000/mm^3^), CRP of 0.45 mg/dL (normal value: <0.5 mg/dL) and PCT of 0.18 ng/mL (normal value: <0.5 ng/mL). Electrolytes, liver, and renal function tests were normal.

The baby received local treatment with a 2% eosin solution and daily application of advanced dressings with silicone and polyurethane foam (Mepilex Lite). Sterile gauze was used to wrap the lesions, and the dressings were changed daily or every two days.

Some lesions on the left leg and the left hand had purulent material, and the skin swab performed was positive for Methicillin-susceptible *Staphylococcus aureus* (MSSA) (Figure 3B,C). Oral therapy with amoxicillin and clavulanic acid was started (the baby initially had no intravenous accesses because of skin fragility) and continued for about 14 days. Urine and blood cultures were negative.

Skin biopsy and genetic tests confirmed the suspicion of epidermolysis bullosa. Immunofluorescence antigen mapping showed a complete absence of laminin 332 (laminin 5, mAb GB3) along the dermal–epidermal membrane zone, and the diagnosis of severe junctional Epidermolysis Bullosa (JEB) was then confirmed by next-generation sequencing, revealing a novel homozygous LAMB3 mutation: c.[298 + 5G > C];[298 + 5G > C].

On the 33rd day of life, the baby presented with fever, irritability, and feeding difficulties. Blood tests showed a whole blood count of 35,000 cells/mm^3^ (64.5% neutrophils and 26% lymphocytes), hemoglobin of 10.6 g/dL, platelet count of 122,000/mm^3^, positive infection biomarkers with CRP of 10.1 mg/dL and PCT of 1.16 ng/mL. A central venous catheter was placed, and intravenous therapy with piperacillin–tazobactam was started. Urine culture was negative and a lumbar punction was not performed. Blood culture was positive for *Staphylococcus aureus* resistant to Penicillin G (i.e., MRSA). Antibiotic therapy was switched to intravenous vancomycin with rapid improvement of general clinical conditions. At discharge from our unit, extensive skin and mucous lesions were still visible on the bilateral lower and upper lips, feet, hands, trunk, sacral area, and face. The baby was transferred back again to the hospital where she was born in Southern Italy to continue antibiotic treatment upon the request of parents who worked and lived there (Table 5).

### 6.2. Management

When assessing a baby with blisters and/or erosions, the differential diagnosis is extensive and ranges from commonly acquired etiologies, such as infection-related blisters (Staphylococcal scalded skin syndrome, herpes simplex, bullous impetigo, candidiasis, varicella, autoimmune blistering disorders) to the rare group of inherited bullous skin disorders known with the term “inherited Epidermolysis Bullosa” (EB), transmitted in an autosomal dominant or recessive manner [1]. Indeed, the newborn baby with EB may present with localized or extensive blistering at delivery or within the first few days of life. Clinical examination can be suggestive for EB, and in some cases, for EB subtype, but immunofluorescence antigen mapping, transmission electron microscopy (in selected cases), and genetic testing are necessary to confirm the diagnosis [24,25].

Based on the level of tissue separation and cleavage planes within the skin basement membrane, four major types of EB are currently described: EB simplex (EBS), junctional EB (JEB), dystrophic EB (DEB), and Kindler EB (KEB). To date, no specific curative treatment exists for EB [26]. The prevention of new blisters development and accurate wound care represent the optimal therapeutic approach. The early care of newborns with EB should occur in a neonatal or pediatric intensive care unit (NICU or PICU, respectively) with multidisciplinary experience, personnel, and resources to address significant erosions or possible problems associated with broad skin erosion, considering that EB acts as a systemic disorder [27].

Our case shows how, in case of fever and/or feeding aversion, an overlapping infection should always be ruled out, with a lower threshold for suspicion and initiation of antibiotic treatment rather than in infants with intact skin. These patients, in particular with severe JEB, are more likely to develop sepsis, with a significant mortality risk. *Staphylococcus aureus* and *Streptococcus pyogenes* are the most commonly involved bacteria; however, infections with gram-negative bacteria (*Pseudomonas* species and *Escherichia coli*) can also occur [28]. Furthermore, the presence of indwelling central venous catheters increases the risk of bloodstream infections by both bacterial and fungal organisms. In addition, the low nutritional status could confer a diminished resistance to infections in these infants [28].

In particular, severe generalized recessive DEB and severe JEB subtypes are associated with immunological problems, such as decreased lymphocyte production [29].

Finally, the impact of colonization should be considered. Skin ulceration in EB patients results in the formation of wounds that become colonized by several bacteria, including *S. aureus*. Over time, within about 2 years, more than 90% of the chronic wounds of EB patients became colonized by multiple types of *S. aureus* [30]. The number of bacteria (bacterial growth greater than 100,000 organisms per gram of tissue), virulence, and pathogenicity of the individual bacteria, and adequate innate and adaptive immune responses of the host influence whether the colonizing organism invades tissues or not [31]. Additionally, Levin et al. reported methicillin resistance and mupirocin resistance in 47% and 40% of EB patients tested, respectively, highlighting the importance of antimicrobial strategies to limit antibiotic resistance [32]. Hence, infection prevention remains the best strategy for EB patients to improve survival and morbidity.

## 7. Burns with Overlapped Staphylococcal Infections

### 7.1. Patient

**Figure 4:**
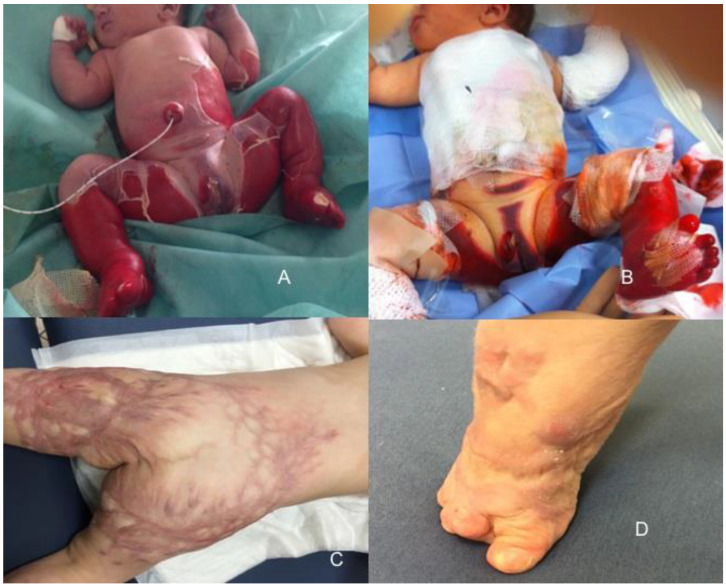
Case of burns in a neonate with overlapped Staphylococcal infection: the skin of more than half of the body was initially injured (**A**), with the second to fourth toes that appeared necrotic (**B**). Wounds healed after 38 days of therapy, with serious scars at six months of life requiring numerous plastic surgical interventions (**C**,**D**).

A 3420 g full-term neonate was born via uncomplicated vaginal delivery and regularly underwent baths at birth. After two hours, she was noted to be suffering from extensive skin de-epithelialization in the lower part of the body. Epidermolysis bullosa was suspected; thus, at 8 h of life, she was referred to our Neonatal Intensive Care Unit (NICU) with spontaneous breathing while receiving an intravenous 10% dextrose solution through the umbilical venous catheter and ampicillin–sulbactam antibiotic empiric therapy. On the clinical examination, the skin of more than half of the body was injured; the skin appeared denuded posteriorly from the subscapular area to the toes and in the front from the navel to the toes, including the external genitalia (Figure 4A). The second to fourth toes appeared necrotic (Figure 4B).

We found markedly increased white cell count (51,240 cells/mm^3^) (normal values: 6000–21,000 cells/mm^3^), low total protein (3.5 g/dL) (normal value: 6–8 g/dL), normal C-reactive protein (0.21 mg/dL) (normal value: <0.5 mg/dL), and initial negative blood and microbiological cultures. Electrolytes, liver, and renal function tests were normal.

Analgesic and supportive therapies were started; intravenous fluid intake with 10% dextrose and electrolytes were administered to reach a urine output target of 1.5 to 3.0 mL/kg/h. The infusion was adjusted according to the baby’s oral intake and urine output. Serum electrolytes and renal function parameters always remained within normal ranges.

Prophylactic intravenous antibiotics (ampicillin and netilmicin) were started since admission; they were administered for a total of 10 days because of a blood culture positive for *Staphylococcus epidermidis* and wound swabs in the diaper area positive for *Escherichia coli*, with progressive improvements of white cell counts. Sterile paraffin tulle occlusive dressings were performed, and the neonate was covered by a sterile cotton cloth. Skin dressings were carried out after administering two drops of morphine (Oramorph^®^ 20 mg/mL solution) by mouth. Exfoliative dermatitis evolved in scabs and eschars without the onset of new lesions, and at 20 days from admission, it was necessary to remove multiple eschars by surgery (Table 6). A skin biopsy was carried out, and the results were consistent with skin injuries caused by thermal damage, probably, due to use of hot water. Wounds healed after 38 days of therapy, with serious scars at six months of life requiring numerous plastic surgical interventions (Figure 4C,D).

### 7.2. Management

Younger children are more likely to sustain injuries from scald burns caused by hot liquids or steam, while older children are more likely to sustain injuries from flame burns caused by fire contact [33]. In neonates, most cases of burns occur in the hospital setting and should be considered in the differential diagnosis of extensive bullous disease [34,35,36]; patient and family history, clinical features, and a skin biopsy may help the diagnosis.

The age at the time of the accident is the main predictor of mortality in burn victims, but its effect in neonates is less clear than in older pediatric patients and adults. Although rare, burns suffered by neonates can be fatal [37]. Rehydration with dextrose solution to maintain adequate urine output and pain management are mandatory in the management of burned neonates.

Infections are a major cause of death in burn patients who survive the first 72 h, and the risk of infections caused by multidrug-resistant bacterial pathogens increases with the hospital length of stay. Burn wounds are initially sterile after the thermal insult, and more sensitive gram-positive organisms prevail in the early days of postburn hospitalization, whereas more resistant gram-negative species are discovered afterward, as in our case [38]. Although antibiotic prophylaxis was ineffective in preventing infections in pediatric burns in most cohorts [39], no studies included only neonates and small infants. Therefore, considering the higher risk of mortality in this fragile category [40], intravenous antibiotics should be considered and stopped if cultures tested negative, reducing as soon as possible the duration of antibiotic therapy [41].

Furthermore, perioperative antibiotic prophylaxis should be administered when surgery is needed [42]. A multidisciplinary approach is highly recommended, including an infectious disease specialist and a plastic surgeon.

## 8. Conclusions

In treating Staphylococcal infections involving skin in neonates, the presence or not of systemic symptoms should be considered. In the lack of evidence-based guidelines in this age category, treatment should be individualized according to several factors including the extension of the disease or further skin comorbidities (such as skin fragility).

In the case of bullous impetigo, topical treatment is always indicated and may be sufficient for limited impetigo extension [14]. However, systemic treatment is especially recommended in neonates with systemic symptoms, with multiple lesions, and/or with parents not able to manage the treatment at home [14]. In the case of SSSS, patients typically require hospital admission for intravenous antibiotic therapy and supportive care.

Concerning epidermolysis bullosa, in case of fever and/or feeding aversion, an overlapping infection should always be ruled out, with a lower threshold for suspicion and initiation of antibiotic treatment rather than in infants with intact skin.

Finally, burns are rare in neonates and small infants, and we have no evidence-based recommendations not to start antibiotics. Given their greater risk of death [40], intravenous antibiotics can be started and terminated as soon as possible if cultures are negative.

When the differential diagnosis and the management of blistering skin diseases are challenging, a multidisciplinary team consisting of a neonatologist, dermatologist, infectious disease specialist, geneticist, psychologist, and nurse should be involved in the management of these complex pathologies, such as in the case of epidermolysis bullosa. An aspect of great importance is that of family, psychological, and logistical support since the management of these diseases can be onerous, even at home, and the aesthetic aspects have considerable emotional impact on parents.

## Figures and Tables

**Table 1:** Summary of case 1 with Staphylococcal bullous impetigo.

**Clinical Manifestations**	Irritability and poor feeding
	Widespread maculopapular lesions with honey-colored crusts
**Examinations**	CRP and PCT: negative
	Skin lesions swabs: positive for *S. aureus* MSSA
**Treatment**	Intravenous ampicillin for 5 days
	Local 2% eosin solution for 5 days

**Table 2:** Our suggested approach to initial antibiotic therapy for suspected Staphylococcal infections in neonates.

Term Neonates		Preterm Neonatesor Small-for-Gestational-Age Neonates (<2500 g)
Without Systemic Findings	With Systemic Findings (Fever or Low Temperature, Ill-Appearance, Poor Feeding…)	
Topical antibiotic therapy for 7–10 days (e.g., fusidic acid),with at least 20 days of close follow-up	Intravenous therapy for 5–7 days (e.g., ampicillin)	Intravenous therapy for 5–7 days (e.g., ampicillin)
	In case of >15% of the community, *S. aureus* isolates are MRSA. An empiric intravenous coverage for MRSA should be considered (e.g., vancomycin, teicoplanin, linezolid or clindamycin)	

**Table 3:** Summary of case 2 with Staphylococcal scalded skin syndrome.

**Clinical Manifestations**	Discomfort
	Skin lesions characterized by honey-colored crusts on the chin and blisters in the axillary region progressively increasing in size
**Examinations**	CRP and PCT: negative
	Skin lesions swabs: positive for *S. aureus* MSSA
**Treatment**	Intravenous ampicillin for 7 days
	Local 2% eosin solution for 5 days

**Table 4:** CDC Case definition for Staphylococcal Scalded Skin Syndrome (SSSS).

CDC Case Definition for SSSS	
Clinical Criteria	- Temperature > 38.9 °C - Diffuse macular erythroderma - Desquamation, 1 to 2 weeks after onset, particularly palmoplantar - Hypotension for age - Multisystem involvement with three or more of the following: Gastrointestinal Muscular Renal Hepatic Hematologic Central nervous system
Laboratory Criteria	Negative test results for the following (if obtained): - Throat, cerebrospinal fluid, blood cultures (although blood may be positive for *S. aureus*) - Serological tests for other micro-organisms (HSV, measles, or others)
✓ Probable” disease: laboratory criteria + 4 out of 5 clinical criteria ✓ Confirmed” disease: laboratory criteria + all 5 clinical criteria (unless patient dies before desquamation)	

**Table 5:** Summary of case 3 with epidermolysis bullosa and Staphylococcal sepsis probably due to extended skin lesions.

**Clinical Manifestations**	Severe Junctional Epidermolysis Bullosa (JEB) with extensive skin and mucous erosions
	Fever, irritability, and feeding difficulties
**Examinations**	CRP and PCT: positive
	Blood culture: positive for *S. aureus* MRSA
**Treatment**	Intravenous vancomycin for 10 days
	Advanced dressings with topical antiseptic agents

**Table 6:** Summary of case 4 with severe burns and sepsis due to *Staphylococcus epidermidis* and *Escherichia coli*.

**Clinical Manifestations**	Skin de-epithelialization in the lower part of the body after baths at birth
	Necrotic toes
**Examinations**	CRP: negative
	Increased white cell count
	Blood culture: positive for *S. epidermidis*
	Wound swabs: positive for *E. coli*
**Treatment**	Intravenous ampicillin and netilmicin for 10 days
	Advanced dressings and remotion of multiple eschars by surgery

## Data Availability

All considered data in this study are reported in this article.
